# Predictive value of early lactate (<6 h) during normothermic machine perfusion and outcome after liver transplantation: results from a multicentre study

**DOI:** 10.1093/bjs/znae084

**Published:** 2024-06-14

**Authors:** Julia Hofmann, Andras T Meszaros, Andrew Butler, Angus Hann, Hermien Hartog, Felicia Kneifel, Satheesh Iype, Keziah Crick, Benno Cardini, Barbara Fiore, Magdy Attia, Joerg-Matthias Pollok, Andreas Pascher, Thomas Vogel, Thamara Perera, Christopher J E Watson, Stefan Schneeberger

**Affiliations:** Department of Visceral, Transplant and Thoracic Surgery, Medical University of Innsbruck, Innsbruck, Austria; Department of Visceral, Transplant and Thoracic Surgery, Medical University of Innsbruck, Innsbruck, Austria; Department of Surgery, University of Cambridge, Cambridge, UK; Liver Unit, Queen Elizabeth Hospital, University Hospitals Birmingham NHS Foundation Trust (UHBFT), Birmingham, UK; Liver Unit, Queen Elizabeth Hospital, University Hospitals Birmingham NHS Foundation Trust (UHBFT), Birmingham, UK; Department of General, Visceral and Transplant Surgery, University Hospital of Münster (UKM), Münster, Germany; Department of HPB and Liver Transplantation, The Royal Free Hospital, Hampstead, London, UK; Department of HPB and Liver Transplantation, The Royal Free Hospital, Hampstead, London, UK; Department of Visceral, Transplant and Thoracic Surgery, Medical University of Innsbruck, Innsbruck, Austria; Liver Transplant Unit, Leeds Teaching Hospitals, NHS Foundation Trust, Leeds, UK; Liver Transplant Unit, Leeds Teaching Hospitals, NHS Foundation Trust, Leeds, UK; Department of HPB and Liver Transplantation, The Royal Free Hospital, Hampstead, London, UK; Division of Surgery and Interventional Science, University College London, London, UK; Department of General, Visceral and Transplant Surgery, University Hospital of Münster (UKM), Münster, Germany; Department of General, Visceral and Transplant Surgery, University Hospital of Münster (UKM), Münster, Germany; Liver Unit, Queen Elizabeth Hospital, University Hospitals Birmingham NHS Foundation Trust (UHBFT), Birmingham, UK; Department of Surgery, University of Cambridge, Cambridge, UK; Department of Visceral, Transplant and Thoracic Surgery, Medical University of Innsbruck, Innsbruck, Austria

## Abstract

**Background:**

Biomarkers with strong predictive capacity towards transplantation outcome for livers undergoing normothermic machine perfusion (NMP) are needed. We investigated lactate clearing capacity as a basic function of liver viability during the first 6 h of NMP.

**Methods:**

A trial conducted in 6 high-volume transplant centres in Europe. All centres applied a back-to-base NMP approach with the OrganOx metra system. Perfusate lactate levels at start, 1, 2, 4 and 6 h of NMP were assessed individually and as area under the curve (AUC) and correlated with EAD (early allograft dysfunction), MEAF (model for early allograft function) and modified L-GrAFT (liver graft assessment following transplantation) scores.

**Results:**

A total of 509 livers underwent ≥6 h of NMP before transplantation in 6 centres in the UK, Germany and Austria. The donor age was 53 (40–63) years (median, i.q.r.).

The total NMP time was 10.8 (7.9–15.7) h. EAD occurred in 26%, MEAF was 4.72 (3.54–6.05) and L-GrAFT_10_ −0.96 (−1.52–−0.32). Lactate at 1, 2 and 6 h correlated with increasing robustness with MEAF. Rather than a binary assessment with a cut-off value at 2 h, the actual 2 h lactate level correlated with the MEAF (*P* = 0.0306 *versus P* = 0.0002, Pearson *r* = 0.01087 *versus r* = 0.1734). The absolute lactate concentration at 6 h, the AUC of 0–6 h and 1–6 h (*P* < 0.0001, *r* = 0.3176) were the strongest predictors of MEAF.

**Conclusion:**

Lactate measured 1–6 h and lactate levels at 6 h correlate strongly with risk of liver allograft dysfunction upon transplantation. The robustness of predicting MEAF by lactate increases with perfusion duration. Monitoring lactate levels should be extended to at least 6 h of NMP routinely to improve clinical outcome.

## Teaser

Biomarkers with strong predictive capacity towards transplantation outcome for livers undergoing normothermic machine perfusion are needed. We investigated lactate clearing capacity as a basic function of liver viability during the first 6 h of NMP in a multicentre trial in 509 livers and found the absolute lactate concentration at 6 h, the AUC of 0–6 h and 1–6 h were the strongest predictors of clinical outcome. Monitoring lactate levels should be extended to at least 6 h of NMP routinely to improve clinical outcome.

## Graphical Abstract

**Graphical Abstract:**
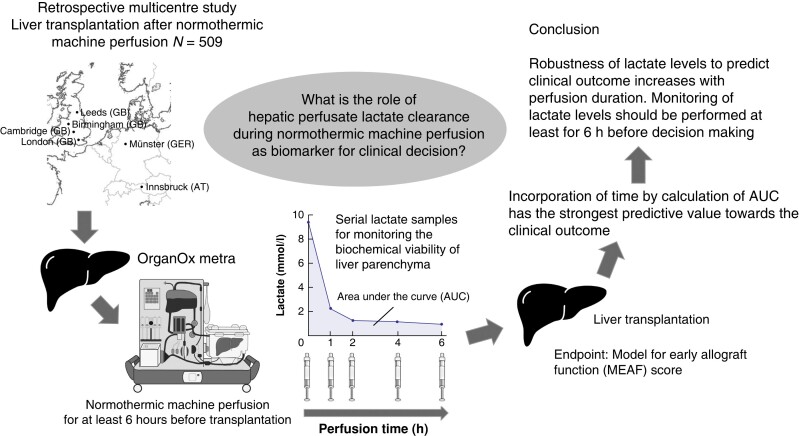

## Introduction

Liver transplantation remains the only curative treatment for end-stage liver disease. However, the demand for organs exceeds the number available. To overcome this limitation, extended criteria donors have been routinely considered for transplantation in recent years^1^. The most common attributes of extended criteria donor livers are advanced donor age, steatosis, donation after circulatory determination of death (DCD), organ dysfunction at procurement and prolonged ICU stay, disease transmission, such as hepatitis B virus and prolonged cold ischaemia time. Although extended criteria donor organs are associated with higher risk of post-transplant dysfunction, careful selection of these organs leads to excellent results^2^. In light of this development, more accurate graft quality and function assessment tools for outcome prediction are warranted. In the past decade, *ex situ* machine preservation techniques have been implemented into the clinical routine in numerous transplant centres. This technology may not only limit the injury induced by ischaemia and reperfusion, but also allow for real-time evaluation of organ and cell function and viability. The latter is a key feature of normothermic machine perfusion (NMP). This perfusion modality enables preservation under close-to-physiological conditions. The hepatic artery and portal vein are cannulated and perfused with an oxygenated, nutrient-rich blood at 37°C. This enables restoration of cellular and metabolic functions^3,4^.

Thus far, various biomarkers analysed in tissue biopsies or samples of the perfusate have been proposed as predictors of the clinical outcome. Watson and Jochmans suggested perfusate glucose, lactate and transaminases as well as bile glucose and pH^5^, Weissenbacher *et al*. found a predictive value for perfusate enzyme levels^6^, whereas others suggest a composite score consisting of biliary bicarbonate, pH and glucose^7^ or a combination of lactate clearance, pH maintenance, bile production, vascular flow patterns and liver macroscopic appearance^8,9^. In the latter study, a cut-off value of perfusate lactate level of <2.5 mmol/l after 2 h of NMP was used. Results from a follow-up study indicate that the period to meet the threshold can be safely extended to 4 h NMP^10^. Another report suggests expanding the preservation time even further^11^. Our group found no significant difference in lactate levels between transplanted and discarded livers at 2 h, whereas a significant difference was seen at 6 h of NMP^12^. Based on the above-mentioned observation, we hypothesized that although lactate clearance may be an appropriate biomarker, the metabolic recovery time with a meaningful predictive value may be longer than 2 h. Hence, we performed a retrospective multicentre study to evaluate the predictive value of lactate levels during the first 6 h of NMP.

## Material and methods

### Patients and trial design

The trial was conducted in 6 high-volume centres in Europe: Department of Visceral, Transplant and Thoracic Surgery, Medical University of Innsbruck, Austria; The Roy Calne Transplant Unit, Cambridge University Hospitals, Cambridge, UK; Liver Unit, Queen Elizabeth Hospital, University Hospitals Birmingham NHS Foundation Trust (UHBFT), UK; Department of General, Visceral and Transplant Surgery, University Hospital of Münster (UKM), Germany; Department of HPB and Liver Transplantation, The Royal Free Hospital, Pond Street, Hampstead, London, UK; Liver Transplant Unit, Leeds Teaching Hospitals, NHS Foundation Trust, Leeds, UK.

Patients of at least 18 years of age who underwent liver transplantation were considered for this retrospective multicentre study. Organs stemmed from donation after brain death (DBD) or donation after circulatory death (DCD) donors and were preserved with normothermic machine perfusion for at least 6 h. A total of 509 patients were enrolled. Patient data were provided as service evaluation^13^.

### Normothermic machine perfusion

The Metra (OrganOx, Oxford, UK) was used for all *ex situ* liver perfusions. Livers were perfused for up to 24 h prior to transplantation. The device set up and perfusions were performed as previously described^4,14,15^. Briefly, after back-table preparation the hepatic artery, portal vein, inferior vena cava and bile duct were cannulated. Livers were perfused at 37°C using packed red blood cells and succinylated gelatin (Gelofusine, B.Braun, Melsungen, Germany) with heparin, insulin, prostacyclin, bile salts and parenteral nutrition added at continuous rate throughout the perfusion. A physiological pH was maintained and, if required, sodium bicarbonate for pH correction was added. During NMP, perfusate samples were collected after start of perfusion (5–15 min), at 1, 2 and 6 h and every 6 h throughout the rest of the run. The samples were then analysed for lactate concentration according to the centres’ practice.

### Clinical endpoints

As primary endpoints, EAD (early allograft dysfunction)^16^, MEAF (model for early allograft function)^17^ and modified L-GrAFT (liver graft assessment following transplantation)^18^ scores were calculated. Because aspartate aminotransferase (AST) was not assessed in some participating centres, alanine aminotransferase (ALT) was used as an alternative for L-GrAFT calculations in all patients (L-GrAFT_ALT_). The 90-day and 1-year patient and graft survival served as secondary endpoints.

### Statistical analysis

Statistical analysis was performed using R Studio 9.1 and GraphPad Prism 9. All variables were tested for normality using the Shapiro–Wilk test. Descriptive statistics are expressed as mean ± s.d. for normal distributed continuous variables and as median and i.q.r. for non-normal distributed continuous variables. Categorical variables are expressed as percentage. For calculation of the area under the curve (AUC) of the lactate levels to include the time component during NMP, the R package ‘stats’ was used. Linear regression was applied to investigate the relation between lactate levels at single time points or AUC values with primary and secondary endpoints. A mixed model for repeated measurements was applied to compare the effect size of the predictive value towards the clinical outcome. All statistical tests with a *P* < 0.050 were considered statistically significant.

## Results

### Donor characteristics and normothermic machine perfusion

A total of 509 livers were included in this multicentre study. All grafts were transplanted following NMP for at least 6 h. The decision for transplantation was based on criteria of the respective transplant centre (*Table S1*). The details of the donor demographics and perfusion characteristics are summarized in *Table 1*. The majority of grafts stemmed from DBD donors whereas 125 (25%) were from DCD donors. The mean donor age was 51 (40–65) years, the median BMI was 26 (23–29) kg/m^2^ and 238 (48%) were female donors. The cold ischaemia time (CIT) was 6.7 (5.6–8.0) h followed by NMP for 10.8 (7.9–15.7) h, resulting in a total preservation time of 17.8 (14.7–22.6) h.

**Table 1:** Donor demographics, recipient demographics and clinical outcome

	*N* = 509
**Donor demographics**	
Donor age (years)*	53 (40–63)
Donor sex ratio (Male:Female)†	263:238 (52:48)
Donor BMI (kg/m^2^)*	26 (23–29)
Donor type (DBD:DCD)†	384:125 (75:25)
Cold ischaemia time (h)*	6.7 (5.6–8.0)
NMP duration (h)*	10.8 (7.9–15.7)
Total preservation time (h)*	17.8 (14.7–22.6)
**Recipient demographics**	
Recipient age (years)*	56 (46–63)
Donor sex ratio (Male:Female)†	248:132 (65:35)
Donor BMI (kg/m^2^)*	27 (23–30)
Recipient labMELD at transplantation†	14 (11–20)
**Clinical outcome**	
90-day patient survival†	472 (93)
90-day graft survival†	472 (93)
1-year patient survival†	456 (90)
1-year graft survival†	457 (90)
MEAF*	4.72 (3.54–6.05)
L-GrAFT 7d*	0.11 (−0.55–0.90)
L-GrAFT 10d*	−0.96 (−1.52–−0.32)
L-GrAFT_ALT_ 7d*	0.42 (−0.36–1.32)
L-GrAFT_ALT_ 10d*	−0.38 (−1.03–0.43)
EAD†	124 (26)

DBD, donation after brain death; DCD, donation after circulatory death; NMP, normothermic machine perfusion; labMELD, model of end-stage liver disease; MEAF, model for early allograft dysfunction; L-GrAFT, liver graft assessment following transplantation; EAD, early allograft dysfunction. *Values are median (i.q.r.). †Values are *N* (%).

### Recipient characteristics and clinical outcome

The detailed recipient characteristics are shown in *Table 1*. The median age of the recipients was 56 (46–63) years, the median BMI was 27 (23–30) kg/m^2^ and 135 (35%) were female. The median labMELD (model of end-stage liver disease) score at the time of transplantation was 14 (11–20). For assessment of the clinical outcome, surrogate parameters indicative for the long-term results were calculated. EAD occurred in 124 (26%) of patients and the median MEAF score was 4.72 (3.54–6.05). The calculated L-GrAFT at day 7 was 0.11 (−0.55–0.90) and −0.96 (−1.52–−0.32) on day 10. L-GrAFT_ALT_ was 0.42 (−0.36–1.32) and −0.38 (−1.03–0.43) on days 7 and 10 respectively. The one-year patient survival rate and the one-year graft survival were 90%.

### Assessment of lactate levels during normothermic machine perfusion

**Fig. 1:**
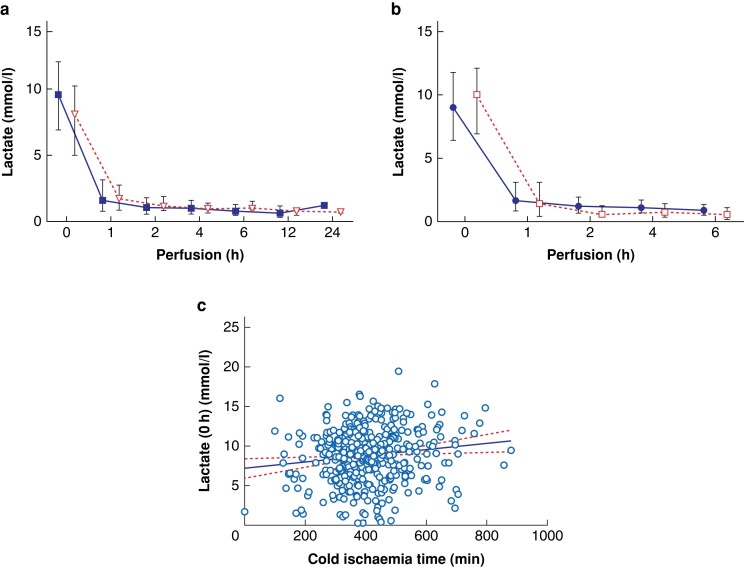
Perfusate lactate levels during normothermic machine perfusion (NMP) for the surviving recipients (solid boxes) and non-surviving (open triangles) (a) and for donation after brain death (solid circles) or donation after circulatory death (open boxes) (b) as mean ± s.d **c** Linear regression analysis of perfusate lactate as a function of cold ischaemia time after start of NMP.

During NMP, perfusate samples were collected and analysed for lactate. All livers showed high lactate levels after start of NMP (*Fig. 1*, *Table S2*). The mean lactate value in samples taken after 5–15 min was 9.38 ± 4.04 mmol/l. Lactate decreased to 2.32 ± 2.23 mmol/l and 1.33 ± 1.07 mmol/l after 1 and 2 h of perfusion respectively. During the subsequent course, the lactate levels decreased further steadily to reach 1.20 ± 0.89 mmol/l at 4 h and 0.98 ± 0.76 mmol/l at 6 h of perfusion, indicating further dynamics after the first 2 h. In total, 86% of the livers met the previously described viability criteria of lactate levels <2.5 mmol/l after 2 h of perfusion^9^. In the follow-up, this cohort reached a one-year patient survival of 90%. Interestingly, livers with lactate levels falling <2.5 mmol/l only at 4 h or 6 h of NMP displayed a one-year patient survival of 89% and 100% respectively (*Fig. S1*). This underlines the possible benefits of prolonged monitoring before decision-making.

**Fig. 2:**
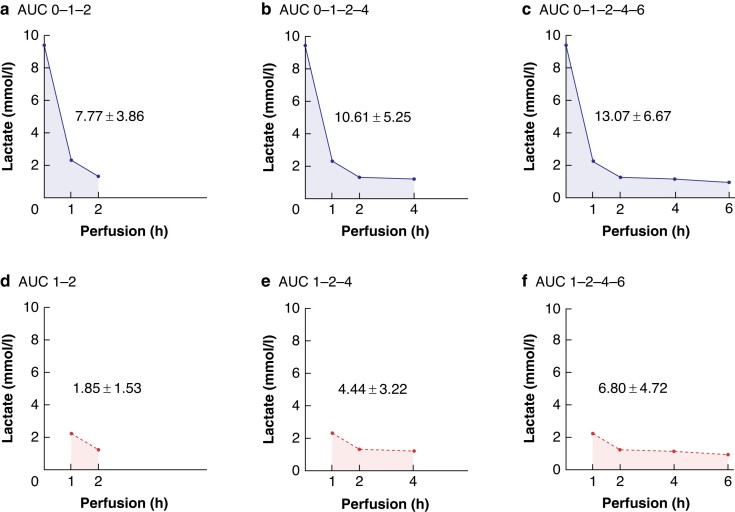
Area under the curve (AUC) calculations AUC was determined with incorporation of the lactate measurements after the start of normothermic machine perfusion (NMP), 1 and 2 h (**a**, AUC 0–1–2), additionally with 4 h (**b**, AUC 0–1–2–4) or 4 and 6 h (**c**, AUC 0–1–2–4–6). AUC was determined with omitting the first lactate measurement after the start of NMP with incorporation of 1 and 2 h measurements (**d**, AUC 1–2), additionally with 4 h (**e**, AUC 1–2–4) or 4 and 6 h (**f**, AUC 1–2–4–6).

In order to incorporate the individual metabolic recovery of the livers, the AUC during the course of NMP was determined. The calculations were performed both including (*Fig. 2a–c*) and omitting (*Fig. 2d–f*) the first lactate measurements after the start of NMP. Moreover, calculations were performed including each of the successive time points 2, 4 and 6 h of perfusion.

The AUC for the observation period between start and 2 h of perfusion was 7.77 ± 3.86 mmol/l × h. The AUC incorporating 4 and 6 h of perfusion were 10.61 ± 5.25 and 13.07 ± 6.67 respectively. The AUC when omitting the first lactate measurement was 1.85 ± 1.53; 4.44 ± 3.22 and 6.80 ± 4.72 for the periods of 2, 4 and 6 h respectively.

In a next step, we performed a subgroup analysis to investigate the impact of donor type (*Fig. 1b*, *Table S2*) and CIT (*Fig. 1c*) on the lactate course. No significant difference between DBD and DCD livers was found for at any of the single time point lactate measurements. However, significantly lower lactate 1 h–2 h–4 h and 1 h–2 h–4 h–6 h AUCs were observed in the DCD group compared to DBD. In addition, a positive correlation for CIT and perfusate lactate concentrations was found.

### Predictive value of lactate levels on the clinical outcome

**Fig. 3:**
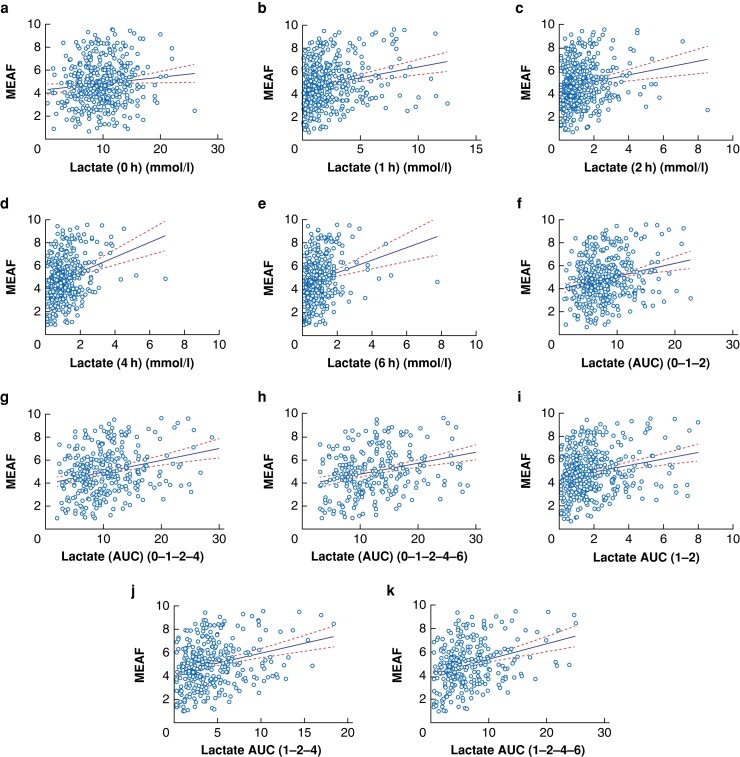
Linear regression analysis against model for early allograft function (MEAF) and the single time point measurements (a–e) and calculated AUCs (f–k) AUC, area under the curve

**Fig. 4:**
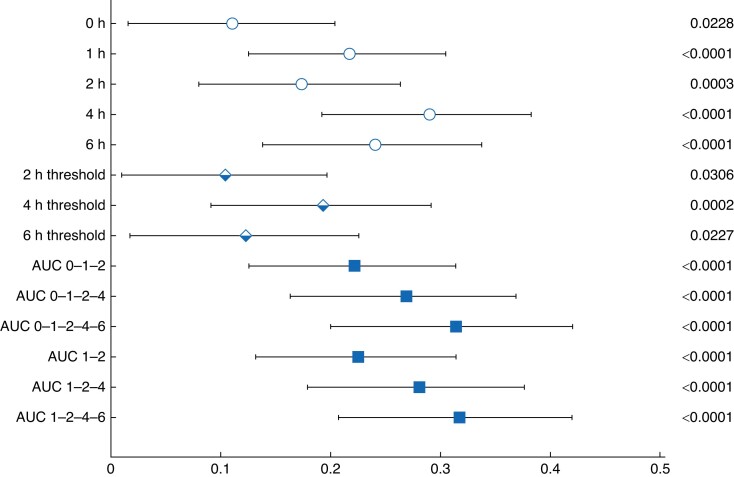
Forest plot of mixed model for repeated measurements Symbols show the effect size along with 95% confidence intervals.

In the next step, we investigated the predictive value of the single time point measurements as well as 2 h, 4 h, 6 h thresholds and AUCs. Thus, we performed a linear regression of MEAF, L-GrAFT_7_, L-GrAFT_10_, L-GrAFT_ALT7_ and L-GrAFT_ALT10_ as a function of perfusate lactate levels. We did not find a correlation for AUCs, single time points or thresholds towards L-GrAFT. However, we found a strong predictive value of lactate towards MEAF. A positive correlation was found for lactate levels at all time points. This correlation reached a strong significance only for time points after 1 h of perfusion. The correlation increased with prolongation of perfusion indicated by a progressively steeper regression line (*Fig. 3a–e*) and by higher Pearson *r* values up to 0.2904 (*Table S3*). The predictive value of lactate AUCs was stronger than the single time point data (*Table S3*, *Figs 3f–k and 4*). For all calculated AUCs, a significant positive correlation and strong predictive values were found, confirmed by a Pearson *r* higher than 0.2 for all AUCs. The strongest predictive value was found for calculations incorporating the 6 h measurement with a Pearson *r* of 0.3146 for AUC 0–1–2–4–6 and Pearson *r* of 0.3176 for AUC 1–2–4–6. In livers with perfusion for extended time periods (>6 h) the predictive capacity of measurements at 12, 18 and 24 h of NMP was analysed. The predictive capacity for the 12 h lactate measurement remained statistically significant (*N* = 111). The number of organs perfused for at least 18 and 24 h was relatively small (*N* = 34 and *N* = 5 respectively). While a correlation remained present, statistical significance was lost. Lactate thresholds were lower compared to respective single time point measurements with Pearson *r* of 0.104 (2 h threshold), 0.194 (4 h threshold) and 0.123 (6 h threshold). We then calculated the predictive value of the different lactate parameters towards the 1-year patient and 1-year graft survival but could not find significant correlations (*Tables S4*, *S5*).

## Discussion

This study represents the largest liver NMP data set published to date and reveals first robust data on a biomarker. All patients undergoing NMP and subsequent liver transplantation in six high-volume NMP centres were included. We could demonstrate that the predictive value of lactate measurements increases with prolongation of NMP, suggesting that monitoring of lactate levels beyond 2 h holds additional value for optimal graft selection.

Together with the implementation of NMP for liver preservation in the clinical routine came the demand for reliable biomarkers aiding the decision-making process. Various parameters have been postulated and investigated in clinical trials. The majority of centres would consider lactate clearance during NMP a critical biomarker; however, the value of lactate has not been formally established in a multicentre study. At the start of NMP, a high lactate concentration is found in the perfusate originating from previously stored packed red blood cells and—to the larger extent—from organ procurement and cold ischaemia-induced disruption of the metabolic capacity of the liver. As a selection criterion for transplantation, Mergental *et al.* suggested a cut-off value of 2.5 mmol/l lactate in the perfusate after 2 h of perfusion^9^. However, this short period for monitoring might not be sufficient because the metabolic recovery rate exhibited large interindividual differences between livers. In a recent study, we could show that intracellular ATP levels in livers increased only marginally at 1 h after start of machine perfusion when compared to the end of static cold storage (SCS), but further increased with prolonged NMP, eventually reaching significance in comparison with SCS^12^. This is in line with a liver NMP study by Raigani *et al.* where they utilized declined human livers^19^. In their study, the energy charge increased significantly 3 h after start of NMP when compared to SCS. Rather than single time point measurements, the AUC of the efficacy of mitochondrial ATP production over 6 h of NMP yielded a predictive value towards the clinical outcome in this study^12^. A recent study by Mergental *et al.* supports the hypothesis of beneficial effects of prolonged monitoring. They transplanted 22 livers that were initially declined but reached the targeted lactate threshold after 4 h^10^. This is in line with reports from Hann *et al.*, who extended the monitoring period of five DBD livers, which had failed to meet the lactate cut-off value after 4 h of perfusion. After reaching the targeted threshold, the livers were successfully transplanted^11^.

As the inclusion criteria were not limited to a certain donor type, we aimed at investigating differences in lactate between DBD and DCD donors. Somewhat surprisingly, we did not find significant differences between the donor types. This might be at least partly attributed to a positive selection bias as DCD organs had a shorter CIT and were younger in our cohort. Indeed, in a previous study, Perera *et al.* found interstitial lactate levels to be significantly higher during SCS in human DCD grafts compared to DBD grafts. In contrast to the present research, the mean donor age was similar for DCD and DBD grafts in their cohort^20^.

Even though the cellular metabolism is significantly decreased during SCS, the metabolic rate remains at about 10% of that at 37°C^21^. Due to the shift to anaerobic metabolism, metabolic products such as lactate accumulate, leading to increasing lactate levels with the prolongation of SCS. In a porcine transplant model with a short CIT (4 h) and prolonged CIT (14 h) group, increased intrahepatic lactate levels during SCS and after reperfusion in the recipient have been found^22^. When we investigated the overall effect of CIT on the perfusate lactate levels after the start of NMP, we found a positive correlation between cold ischaemic time with the initial lactate value.

The correlation of single time point lactate measurements with the MEAF score increased over NMP time. To address individual metabolic recovery, we calculated the AUC for different observation periods. In line with trends observed for single time point measurements, the AUCs incorporating lactate levels up to 6 h NMP revealed the best prediction. The AUC omitting the first lactate measurement (AUC 1–2–4–6) showed a superior correlation compared to the AUC 0–1–2–4–6. Our findings indicate that lactate early after initiation of NMP does not have much value and could be omitted.

Despite the very strong correlation between lactate and MEAF, we did not find a strong correlation between lactate and the 1-year patient and the 1-year graft survival. After the first 90 days, the post-transplant period is increasingly determined by unrelated factors such as recipient factors, immunological factors and patient management in addition to the quality of donor graft^23^. In addition, the validation of surrogate parameters and risk scores in the context of NMP and liver transplantation is pending. Importantly, we found a 1-year patient and 1-year graft survival of 100% for those livers that started clearing lactate only beyond 4 h of NMP, highlighting the great suitability of such organs for transplantation.

Given the complexity of liver pathophysiology during organ retrieval, preservation and transplantation, it is fair to assume that perfusate lactate levels are not the sole biomarker for the outcome upon transplantation. We expect scoring systems composed by various biomarkers including but not limited to lactate to emerge in the future.

The robustness of lactate levels during NMP as biomarker towards the early clinical outcome has been established in this large multicentre trial. Further to the value of lactate, our findings indicate that the time for graft monitoring should be extended to at least 6 h of NMP. The participating centres of the multicentre study have adopted the findings of this study in their machine perfusion routine. Because monitoring of lactate levels is based on point-of-care analyses, such measurements are simple to implement. Adopting this assessment method may help to select organs suitable for transplantation and increase the number of liver grafts.

## Supplementary Material

znae084_Supplementary_Data

## Data Availability

Data supporting the figures and tables of this manuscript are available from the corresponding author upon reasonable request. Aspects of the present work have been presented to: ILTS Annual Congress, Istanbul, Turkey 2022 (oral presentation); Austrotransplant, Mayrhofen, Austria, 2022 (oral presentation); ESOT Congress, Athens, Greece, 2023 (oral presentation).
