# Erratum to: Predictive value of early lactate (<6 h) during normothermic machine perfusion and outcome after liver transplantation: results from a multicentre study

**DOI:** 10.1093/bjs/znae182

**Published:** 2024-07-15

This is an erratum to: Julia Hofmann, Andras T Meszaros, Andrew Butler, Angus Hann, Hermien Hartog, Felicia Kneifel, Satheesh Iype, Keziah Crick, Benno Cardini, Barbara Fiore, Magdy Attia, Joerg-Matthias Pollok, Andreas Pascher, Thomas Vogel, Thamara Perera, Christopher J E Watson, Stefan Schneeberger, Predictive value of early lactate (<6 h) during normothermic machine perfusion and outcome after liver transplantation: results from a multicentre study, *British Journal of Surgery*, Volume 111, Issue 6, June 2024, znae084, https://doi.org/10.1093/bjs/znae084

In the originally published version of this paper a publication error meant that the title was incorrectly given as “Predictive value of early **postoperative** lactate (<6 h) during normothermic machine perfusion and outcome after liver transplantation: results from a multicentre study”. The authors of this study analyzed lactate prior to transplantation so the corrected title is now “Predictive value of early lactate (<6 h) during normothermic machine perfusion and outcome after liver transplantation: results from a multicentre study”.

In addition to this error the “Lay summary” was also not included in the originally published version of this. This has now been restored.

The publisher apologizes to the authors for these errors, and to readers for any inconvenience caused.

