# Supplementary material for: Predictive value of early lactate (<6 h) during normothermic machine perfusion and outcome after liver transplantation: results from a multicentre study
Source: Br J Surg. 2024 Jun 14;111(6):znae084. doi: 10.1093/bjs/znae084 (PMC11177788; doi:10.1093/bjs/znae084)
Supplement: znae084_Supplementary_Data [file znae084_supplementary_data.docx]

**Lactate AUC of 0-6 h during normothermic machine perfusion has strong predictive value towards the outcome after liver transplantation: Results from a multicenter study**

Julia Hofmann^1*^, Andras T. Meszaros^1*^, Andrew Butler^2^, Angus Hann^3^, Hermien Hartog^3^, Felicia Kneifel^4^, Satheesh Iype^5^, Keziah Crick^5^, Benno Cardini^1^, Barbara Fiore^6^, Magdy Attia^6^, Joerg-Matthias Pollok^5,7^, Andreas Pascher^4^, Thomas Vogel^4^, Thamara Perera^3^, Christopher J. E. Watson^2^, Stefan Schneeberger^1^

^1^ Department of Visceral, Transplant and Thoracic Surgery, Medical University of Innsbruck, Innsbruck, Austria

^2^ Department of Surgery, University of Cambridge, Cambridge, United Kingdom

^3^ Liver Unit, Queen Elizabeth Hospital, University Hospitals Birmingham NHS Foundation Trust (UHBFT), Birmingham, United Kingdom

^4^ Department of General, Visceral and Transplant Surgery, University Hospital of Münster (UKM), Münster, Germany

^5^ Department of HPB and Liver Transplantation, The Royal Free Hospital, Pond Street, Hampstead, London, United Kingdom

^6^ Liver Transplant Unit, Leeds Teaching Hospitals, NHS Foundation Trust, Leeds, United Kingdom

^7^ Division of Surgery and Interventional Science, University College London, London, United Kingdom

* contributed equally to this work

**Corresponding author:** Stefan Schneeberger; stefan.schneeberger@i-med.ac.at; Department of Visceral, Transplant and Thoracic Surgery, Medical University of Innsbruck, Anichstrasse 35, 6020 Innsbruck, Austria; +43 512 50422601

**ORCID ID:** 0000-0002-2619-8639**; X:** @schneest

**Supplementary Materials - Index**

| **Supplementary Figures and Tables** |  |
| --- | --- |
| Table S1: Center-specific criteria for decision-making during NMP | *pag. 2* |
| Table S2: Repeated lactate measurements and calculated AUCs | *pag. 3* |
| Figure S1: Survival stratified by the duration of NMP after which the lactate levels reached < 2.5 mmol/L | *pag. 4* |
| Table S3: Correlation of lactate values with MEAF | *pag. 5* |
| Table S4: Correlation of lactate values with the 1-year patient survival | *pag. 6* |
| Table S5: Correlation of lactate values with the 1-year graft survival | *pag. 7* |
| **References** | *pag. 8* |
|  |  |

**Supplementary Figures and Tables**

Table S1: Center specific criteria for decision making during NMP

| **Transplant center** | **Criteria for transplantation during NMP** | **Reference** |
| --- | --- | --- |
| Medical University of Innsbruck | 1. Prompt decline of perfusate lactate levels ≤ 2.5 mmol/L 2. Maintenance of physiological perfusate pH levels (7.30–7.45) without repeated sodium bicarbonate addition 3. Highly elevated tissue damage markers in the perfusate (AST > 20,000 U/L, ALT > 20,000 U/L, LDH > 20,000 U/L) 4. Excessively high IL-6 levels urging for caution | ^1^ |
| University of Cambridge | 1. Continuing requirement for bicarbonate to maintain perfusate pH > 7.2 beyond 2 h. 2. If perfusate glucose < 10 mmol/L at 30 min, a 5-g dextrose challenge should be given. The glucose rate of fall should be ≥ 1 mmol/L/h by 4 h 3. 2 h perfusate lactate < 2.8 mmol/L 4. Perfusate ALT < 6000 U/L at 2 h 5. Maximum bile pH > 7.6 6. Bile glucose ≥10 mmol less than perfusate or, if perfusate glucose <11 mmol/L, a bile glucose ≤2 mmol/L | ^2^ |
| University Münster (UKM) | 1. Perfusate lactate levels were tested 15 min after beginning perfusion and hourly thereafter. | ^3^ |
| Royal Hospital London | 1. Perfusate lactate < 2 mmol/L within 4 h 2. Perfusate ALT < 6000 U/L at 2 h 3. Perfusate pH > 7.3 within 4 h (not requiring additional HCO_3_ after 4 h) 4. Perfusate glucose: evidence of glucose metabolism by decreasing trend of glucose within 4 h (without additional insulin) 5. Bile pH > 7.6 within 4 h |  |
| University of Birmingham | 1. Perfusate lactate ≤ 2.5 mmol/L *(mandatory)* 2. *AND* two or more of the following within 4 h^*^ of starting perfusion must be fulfilled:    1. Perfusate pH ≥ 7.30    2. Evidence of bile production    3. Evidence of metabolism of glucose    4. Stable hepatic artery flow ≥ 150 mL/min and portal venous flow ≥ 500 mL/min    5. Homogenous perfusion   * extension of lactate clearance time to 6 h if failed after 4 h | ^4, 5^ |
| University Leeds | 1. Perfusate lactate ≤ 2.5 mmol/L *(mandatory)* 2. *AND* two or more of the following within 4 h of starting perfusion must be fulfilled:    1. Perfusate pH ≥ 7.30    2. Evidence of bile production    3. Evidence of metabolism of glucose    4. Stable hepatic artery flow ≥ 150 mL/min and portal venous flow ≥ 500 mL/min    5. Homogenous perfusion | ^4^ |

Table S2: Repeated lactate measurements and calculated AUCs

| Perfusate lactate^+^ | Overall cohort | DBD | DCD | *p*-value |
| --- | --- | --- | --- | --- |
| 0 h [mmol/L] | 9.38 ± 4.04 | 9.30 ± 4.16 | 9.61 ± 3.72 | 0.6891 |
| 1 h [mmol/L] | 2.32 ± 2.23 | 2.25 ± 1.91 | 2.51 ± 2.98 | 0.8996 |
| 2 h [mmol/L] | 1.33 ± 1.07 | 1.47 ± 1.08 | 0.91 ± 0.92 | 0.0898 |
| 4 h [mmol/L] | 1.20 ± 0.89 | 1.27 ± 0.88 | 1.00 ± 0.89 | 0.8624 |
| 6 h [mmol/L] | 0.98 ± 0.76 | 1.04 ± 0.75 | 0.79 ± 0.75 | 0.9241 |
| 12 h [mmol/L] | 0.95 ± 0.92 | 0.95 ± 0.94 | 0.95 ± 0.84 | >0.9999 |
| 18 h [mmol/L] | 0.84 ± 0.69 | 0.86 ± 0.70 | 0.67 ± 0.62 | 0.9997 |
| 24 h [mmol/L] | 1.00 ± 0.45 | 1.00 ± 0.45 | n.a. | n.a. |
| AUC 0-1-2 [mmol/L×h] | 7.77 ± 3.86 | 7.76 ± 3.59 | 7.78 ± 4.52 | >0.9999 |
| AUC 0-1-2-4 [mmol/L×h] | 10.61 ± 5.25 | 10.87 ± 5.00 | 9.92 ± 5.83 | 0.6760 |
| AUC 0-1-2-4-6 [mmol/L×h] | 13.07 ± 6.67 | 13.68 ± 6.32 | 11.62 ± 7.28 | 0.1622 |
| AUC 1-2 [mmol/L×h] | 1.85 ± 1.53 | 1.89 ± 1.41 | 1.73 ± 1.83 | 0.9549 |
| AUC 1-2-4 [mmol/L×h] | 4.44 ± 3.22 | 4.71 ± 3.13 | 3.70 ± 3.37 | 0.0761 |
| AUC 1-2-4-6 [mmol/L×h] | 6.80 ± 4.72 | 7.34 ± 4.54 | 5.42 ± 4.92 | 0.0171 |

^+^ values are mean ± SD, Two-way ANOVA with Sidak’s multiple comparisons was applied

Figure S1: Survival stratified by the duration of NMP after which the lactate levels reached < 2.5 mmol/L

Table S3: Correlation of lactate values with MEAF

| Lactate *vs* MEAF | *p*-value | Pearson r |
| --- | --- | --- |
| 0 h^+^ | 0.0228 | 0.1108 |
| 1 h^+^ | <0.0001 | 0.2172 |
| 2 h^+^ | 0.0003 | 0.1734 |
| 4 h^+^ | <0.0001 | 0.2904 |
| 6 h^+^ | <0.0001 | 0.2407 |
| 12 h^+^ | 0.0208 | 0.1945 |
| 18 h^+^ | 0.4063 | 0.1226 |
| 24 h^+^ | 0.5460 | 0.2781 |
| AUC 0-1-2^+^ | <0.0001 | 0.2220 |
| AUC 0-1-2-4^+^ | <0.0001 | 0.2694 |
| **AUC 0-1-2-4-6^+^** | **<0.0001** | **0.3146** |
| AUC 1-2^+^ | <0.0001 | 0.2250 |
| AUC 1-2-4^+^ | <0.0001 | 0.2813 |
| **AUC 1-2-4-6^+^** | **<0.0001** | **0.3176** |
| 2 h threshold* | 0.0306 | 0.1043 |
| 4 h threshold* | 0.0002 | 0.1937 |
| 6 h threshold* | 0.0227 | 0.1230 |

^+^ for continuous variables Pearson correlation was performed, * for categorial variable a point-biserial correlation was performed

Table S4: Correlation of lactate values with the 1-year patient survival

| Lactate *vs* 1-year patient survival | *p*-value | Pearson r |
| --- | --- | --- |
| 0 h^+^ | 0.8141 | 0.0128 |
| 1 h^+^ | 0.1791 | 0.0714 |
| 2 h^+^ | 0.6231 | -0.0262 |
| 4 h^+^ | 0.9653 | 0.0026 |
| 6 h^+^ | 0.3183 | 0.0608 |
| AUC 0-1-2^+^ | 0.5619 | 0.0330 |
| AUC 0-1-2-4^+^ | 0.8124 | 0.0155 |
| AUC 0-1-2-4-6^+^ | 0.7425 | 0.0241 |
| AUC 1-2^+^ | 0.4850 | 0.0383 |
| AUC 1-2-4^+^ | 0.8973 | 0.0081 |
| AUC 1-2-4-6^+^ | 0.7870 | 0.0189 |
| 2 h threshold* | 0.3423 | 0.0457 |
| 4 h threshold* | 0.5074 | 0.0397 |
| 6 h threshold* | 0.4325 | -0.0489 |

^+^ for continuous variables Pearson correlation was performed, * for categorial variable a point-biserial correlation was performed

Table S5: Correlation of lactate values with the 1-year graft survival

| Lactate *vs* 1-year graft survival | *p*-value | Pearson r |
| --- | --- | --- |
| 0 h^+^ | 0.4177 | 0.0442 |
| 1 h^+^ | 0.0434 | 0.1073 |
| 2 h^+^ | 0.5148 | -0.0347 |
| 4 h^+^ | 0.9242 | -0.0056 |
| 6 h^+^ | 0.3213 | 0.0605 |
| AUC 0-1-2^+^ | 0.1349 | 0.0850 |
| AUC 0-1-2-4^+^ | 0.3123 | 0.0660 |
| AUC 0-1-2-4-6^+^ | 0.3117 | 0.0740 |
| AUC 1-2^+^ | 0.2108 | 0.0686 |
| AUC 1-2-4^+^ | 0.7305 | 0.0216 |
| AUC 1-2-4-6^+^ | 0.5646 | 0.0403 |
| 2 h threshold* | 0.3423 | 0.0487 |
| 4 h threshold* | 0.5074 | 0.0397 |
| 6 h threshold* | 0.4325 | -0.0489 |

^+^ for continuous variables Pearson correlation was performed, * for categorial variable a point-biserial correlation was performed

**References**

1. Cardini B, Oberhuber R, Fodor M, Hautz T, Margreiter C, Resch T, Scheidl S, Maglione M, Bosmuller C, Mair H, Frank M, Augustin F, Griesmacher A, Schennach H, Martini J, Breitkopf R, Eschertzhuber S, Pajk W, Obwegeser A, Tilg H, Watson C, Ofner D, Weissenbacher A, Schneeberger S. Clinical Implementation of Prolonged Liver Preservation and Monitoring Through Normothermic Machine Perfusion in Liver Transplantation. *Transplantation* 2020;**104**(9): 1917-1928.

2. Watson CJE, Gaurav R, Fear C, Swift L, Selves L, Ceresa CDL, Upponi SS, Brais R, Allison M, Macdonald-Wallis C, Taylor R, Butler AJ. Predicting Early Allograft Function After Normothermic Machine Perfusion. *Transplantation* 2022;**106**(12): 2391-2398.

3. Becker F, Kneifel F, Riegel A, Katou S, Wagner T, Flammang I, Juratli M, Vogel T, Radunz S, Morgul H, Pascher A, Houben P, Brockmann JG. Ex situ arterial reconstruction prior normothermic machine perfusion of liver grafts. *Langenbecks Arch Surg* 2022;**407**(8): 3833-3841.

4. Mergental H, Laing RW, Kirkham AJ, Perera M, Boteon YL, Attard J, Barton D, Curbishley S, Wilkhu M, Neil DAH, Hubscher SG, Muiesan P, Isaac JR, Roberts KJ, Abradelo M, Schlegel A, Ferguson J, Cilliers H, Bion J, Adams DH, Morris C, Friend PJ, Yap C, Afford SC, Mirza DF. Transplantation of discarded livers following viability testing with normothermic machine perfusion. *Nat Commun* 2020;**11**(1): 2939.

5. Hann A, Lembach H, Nutu A, Mergental H, Isaac JL, Isaac JR, Oo YH, Armstrong MJ, Rajoriya N, Afford S, Bartlett D, Mirza DF, Hartog H, Perera M. Assessment of Deceased Brain Dead Donor Liver Grafts via Normothermic Machine Perfusion: Lactate Clearance Time Threshold Can Be Safely Extended to 6 Hours. *Liver Transpl* 2021.
